# Effects of Psychological Distress and Coping Resources on Internet Gaming Disorder: Comparison between Chinese and Japanese University Students

**DOI:** 10.3390/ijerph19052951

**Published:** 2022-03-03

**Authors:** Anise M. S. Wu, Mark H. C. Lai, Mengxuan Zhang, Masao Yogo, Shu M. Yu, Sijie Mao, Juliet Honglei Chen

**Affiliations:** 1 Department of Psychology, Faculty of Social Sciences, University of Macau, Macao, China; anisewu@um.edu.mo (A.M.S.W.); zhangmengxuan@seu.edu.cn (M.Z.); mogu.yu@unigib.edu.gi (S.M.Y.); sb22218@um.edu.mo (S.M.); 2 Centre for Cognitive and Brain Sciences, Institute of Collaborative Innovation, University of Macau, Macao, China; 3 Department of Psychology, University of Southern California, Los Angeles, CA 90007, USA; hokchiol@usc.edu; 4 Department of Medical Humanities, School of Humanities, Southeast University, Nanjing 211189, China; 5 Faculty of Psychology, Doshisha University, Kyoto 610-0394, Japan; myogo@mail.doshisha.ac.jp; 6 Centre of Excellence in Responsible Gaming, University of Gibraltar, Gibraltar GX11 1AA, Gibraltar

**Keywords:** Internet gaming, psychological distress, depression, anxiety, stress, mindfulness, social support, coping flexibility, cross-cultural

## Abstract

The high prevalence of Internet gaming disorder (IGD) among Asian youth indicates an urgent need to identify protective factors and examine their consistency across Asian cultures in order to facilitate cost-effective interventions. Based on the transactional theory of stress and coping, this study collected data of 1243 online gamers (45% males; 18–25 years) through an anonymous survey from universities in China and Japan and investigated whether three coping resources (i.e., mindfulness, coping flexibility, and social support) serve to protect Chinese and Japanese youth from the impact of psychological distress on IGD tendency. After adjusting for the measurement non-invariance across samples, we found that Japanese students reported higher levels of IGD tendency and psychological distress than Chinese students. The results of multiple-group SEM analyses showed that, after controlling for other predictors, mindfulness served as the strongest protective factor against IGD across samples. Moreover, the buffering effect of mindfulness on the association between psychological distress and IGD tendency of female (but not male) students was observed. Our findings highlighted the cross-cultural invariance of the impact of psychological distress and coping resources on IGD in Chinese and Japanese youth, which can be considered in future IGD prevention programs.

## 1. Introduction

Internet gaming disorder (IGD) appears to be most prevalent among young males in Asian countries [1,2]. The prevalence ranged from 4.6–14.8% [3,4,5,6,7] and 3.3–18.7% (based on a broader screening for Internet addiction) [8,9,10,11,12] among Chinese and Japanese youth, respectively. Previous studies have shown that not only prevalence but also associated factors of Internet-related addictions vary across countries and cultural groups. For example, problematic use of the Internet or social networking sites was consistently associated with emotional stability and depression across cultures [12,13], but with self-esteem in only Bulgarian, German, and the Colombian (but not Spanish) samples [14]. However, little is known regarding invariance of those factors across Asian countries despite the cross-nationally high IGD prevalence. To address this missing link, we aimed to perform intensive empirical examinations on the cross-cultural invariance of salient risk and protective factors for IGD among Chinese and Japanese youth to reveal inter-Asian cultural variance, if any.

As countries that hosted the largest gaming populations in Asia [15], China and Japan both face a long-standing challenge of IGD prevention, especially among the young population. Although these two countries have a shared cultural influence of Confucianism [16,17], one cannot simply assume a common underlying mechanism of IGD among Chinese and Japanese gamers without thorough empirical investigations, considering their intertwined cross-cultural similarities and differences. For example, China and Japan are both more on the collectivism side of the individualism-collectivism continuum, but the former is more collective than the latter on the country level [18,19]. Uncovering the cross-cultural consistency and variations of IGD-related factors in these two countries can shed light on the extent of between-country generalization of IGD preventive measures and enhance the effectiveness and efficiency of IGD prevention across cultures. Therefore, this study specifically focused on testing the cross-cultural invariance of key constructs that displayed promising potentials for understanding gamers’ proneness to IGD in China and Japan to bridge the shortage of such studies in the extant literature.

One of the most salient risk factors of IGD is psychological distress. Gaming often serves as a passive coping strategy against unpleasant emotions and/or real-life problems [20,21]. Disordered gaming is a plausible result of maladaptive coping of psychological distress [22]. Empirical studies have consistently reported that higher levels of psychological distress were positively associated with IGD [3,23,24,25]. In particular, escapism or avoidance, which are positively associated with IGD tendency [26,27], are often reported as a major motive for online gaming among addicts [28,29]. In order to break the vicious cycle of psychological distress and IGD, this study aimed to investigate the protective effect of coping resources on the relationship between psychological distress and IGD tendency among Asian youth within the framework of Lazarus and Folkman’s [30] transactional theory of stress and coping.

The transactional theory of stress and coping contends that the transactions between individuals and the environment determine their capacity to cope and adjust to challenges and threats [30]. In the transactions, cognitive appraisals are activated to evaluate the specific external and internal demands and guide one’s emotional and behavioral responses. During cognitive appraisal processes, an individual identifies the problem, evaluates the degree of the demands, and then considers his/her available resources and coping options. These evaluative processes lead to his/her coping responses. Under this theoretical framework, disordered gaming can be viewed as a maladaptive coping when the individual fails to identify sufficient coping resources to meet the demands (e.g., psychological distress) in the face of stressors. In contrast, effective coping often involves an active, positive, and mutable appraisal process of demands and available coping resources [30,31]. Recent positive psychology studies have identified personal and social resources such as coping flexibility, mindfulness, and social support, which may promote positive coping of distress and enhance psychosocial wellbeing [32,33]. In the gaming context, we expected these personal and social resources to outperform the demands during the appraisal processes and then gamers can choose alternative, more adaptive coping approaches instead of excessive gaming; however, empirically, their relationship with IGD has been rarely examined and compared.

Coping flexibility refers to the ability to effectively modify one’s coping responses according to the nature of a demanding situation [34]. People with higher coping flexibility are more likely to reappraise their coping outcomes and resources and make adjustments for a better outcome. As expected, coping flexibility was negatively associated with mental health problems such as burnout and Internet addiction among Chinese university students [35,36]. In addition to its direct effect, another Chinese longitudinal study has further indicated that coping flexibility might also reduce the adverse effects of emotional distress on disordered Internet gaming tendencies [37]. It is plausible that people with higher coping flexibility are less likely to keep adopting negative or avoidant coping strategies (e.g., gaming) even under psychological distress. In light of that research, both direct and moderating effects of coping flexibility against IGD deserve further empirical investigation.

Mindfulness is defined as one’s capability to pay open, nonjudgmental attention to the experience of the present moment [38]. It facilitates emotional regulation and cognitive appraisal of a problem [39]. It also allows a type of decentering that enables an individual to reappraise adverse events and experiences [40]. Extant cross-sectional empirical studies have consistently reported its negative correlation with not only psychological distress [39,41] but also behavioral addictions, such as problematic Internet use and pathological gambling [42,43,44,45]. Furthermore, Ortner and colleagues [46] provided experimental evidence that mindfulness attenuates the effect of unpleasant stimuli on emotional wellbeing; however, its buffering effect on the relationship between psychological distress and addiction has not been empirically tested.

Appraisal processes always involve evaluating available resources for coping, in which social support is a crucial resource. Perceived social support was shown to be negatively correlated with both psychological distress [47,48] and IGD [6,49]. It also acts as a buffer against adverse events or experiences [50,51,52] and reduces the deleterious effect of psychological distress on substance use [53,54] and online social networking use [55]. In contrast, it is worth noting that Reinecke’s [20] study provided only partial support to the buffering effect of this resource, finding that people who perceived more social support were less likely than their counterparts to use gaming to recuperate from work-related fatigue. To our knowledge, such buffering effects on the relationship between generalized psychological distress and IGD have not been tested.

In summary, the present study aims to explore the cultural (in)variance of both the direct and buffering effects of three coping resources (i.e., coping flexibility, mindfulness, and social support) on IGD among Japanese and Chinese university students. We especially targeted university students because their developmental characteristics and ready access to the Internet make them particularly vulnerable to IGD [56]. Given the different language versions of questionnaires (i.e., Chinese and Japanese) were used to collect data in two countries, our first objective is to establish the measurement invariance of the psychological measures [57] across countries to lay the foundation for subsequent cross-cultural comparisons. Our second objective is to test the cross-cultural invariance of two IGD-based hypotheses under the transactional theory of stress and coping. Specifically, we hypothesized that both psychological distress and IGD tendency are negatively associated with three coping resources, namely, coping flexibility, mindfulness, and social support (Hypothesis 1 [H1]). We also hypothesized that these three coping resources would attenuate the positive relationship between psychological distress and IGD (Hypothesis 2 [H2]). To our knowledge, this study was the first to investigate the potential moderating role of these coping resource variables on IGD in a cross-cultural setting. We strived to unveil the cross-cultural consistency and variance of IGD correlates between Chinese and Japanese young adults and shed light on potential buffers that attenuate the chain of psychological distress and IGD.

## 2. Methods

### 2.1. Procedures and Participants

We surveyed 662 Chinese (*n* = 221 for males, *n* = 441 for females) and 581 Japanese (*n* = 265 for males, *n* = 316 for females) university students who had online gaming experience via the participant pools of universities in China and Japan. The mean ages of our Chinese and Japanese participants were 19.44 and 20.07 years, respectively (range = 18–25). The two samples had no significant difference on study year (Welch *t*[1225.2] = 1.04, *p* = 0.30), but the Chinese sample was significantly younger (Welch *t*[1224.2] = 8.64, *p* < 0.001) and had a significantly higher proportion of females (χ^2^[1] = 19.43, *p* < 0.001). Therefore, the effects of sex and age were controlled in the subsequent analyses, when applied, for cross-cultural comparison.

The data was collected in the same way in China and Japan. We adopted the convenience sampling method to recruit eligible participants (i.e., aged 18 years or above, Chinese/Japanese ethnicity, and with online gaming experience) in courses that were open to students of all majors. Every participant received a briefing about the study’s objectives and participants’ rights prior to data collection. Only those who gave their written consent to participate were invited to voluntarily complete the anonymous survey. Ethics approval was obtained from the departmental ethics committee of the affiliated institute of the corresponding author.

### 2.2. Measures

Based on the literature, we specifically selected five validated inventories for the major variables involved in this study. These measures demonstrated good psychometric properties in previous studies among similar populations as the present study [3,23,35,45,58,59,60,61,62,63,64,65], indicating sufficient suitability to respond to the objectives and questions under investigation. A higher scale score represents a higher level of the corresponding construct. Background information, including sex (1 = *male*, 2 = *female*), age, and study year, were also collected.

#### 2.2.1. IGD Tendency

We used the nine core symptoms of IGD stated in the fifth edition of the *Diagnostic and Statistical Manual of Mental Disorders* [66] for assessing the IGD tendency. The respondents were asked to rate on a 5-point Likert scale, in which 1 = *Never* and 5 = *Very often*, to indicate how frequently they experienced each of these symptoms (e.g., preoccupation with gaming) in the past 12 months. Its Cronbach’s alpha was 0.86 in both Chinese and Japanese samples.

#### 2.2.2. Psychological Distress

Self-reported psychological distress was assessed by the three 7-item subscales (i.e., Depression, Anxiety, and Stress) of the 21-item Depression Anxiety Stress Scales (DASS-21) [67]. Items were rated on a 4-point Likert scale, in which 0 = *Did not apply to me at all* and 3 = *Applied to me very much or most of the time*. A sample item is “I could not seem to experience any positive feeling at all”. The Cronbach’s alpha of the overall scale was 0.93 in both Chinese and Japanese samples.

#### 2.2.3. Trait Mindfulness

The 15-item Mindful Attention Awareness Scale [41] was used to assess trait mindfulness, with a sample item being “I find it difficult to stay focused on what is happening in the present.”. Items were rated on a 5-point Likert scale (1 = *Strongly disagree* to 5 = *Strongly agree*) and then reversed during the scoring phase to make sure a higher score corresponds to a higher level of mindfulness. In this study, the Cronbach’s alpha of this scale was 0.85 in the Chinese sample and 0.84 in the Japanese sample.

#### 2.2.4. Coping Flexibility

The 10-item Coping Flexibility Scale [34] was used to assess participants’ coping flexibility (e.g., “When stressed, I use several ways to cope and make the situation better.”). It has a 4-point Likert response scale, in which 1 = *Not applicable* and 4 = *Very applicable*. Its Cronbach’s alpha was 0.79 and 0.83 in the Chinese and Japanese samples, respectively.

#### 2.2.5. Social Support

Perceived social support was measured by the 12-item Social Support Scale [68], rated and scored on a 5-point Likert scale, in which 1 = *Strongly disagree* to 5 = *Strongly agree*. A sample item is “there is a special person who is around when I am in need”. This study found its Cronbach’s alpha to be 0.93 and 0.92 in the Chinese and Japanese samples, respectively.

### 2.3. Data Analysis

#### 2.3.1. Measurement Invariance and Item Parceling

As this study involved cross-cultural comparisons with participants responding to questionnaires in a different language (i.e., Chinese and Japanese), we first examined measurement invariance of the psychological measures [57] to serve the first study objective. We performed a series of multiple-group confirmatory factor analyses (CFA) using Mplus 7.4 [69] with robust weighted least squares estimation (WLSMV) for ordered categorical items. The models were evaluated using the scaled χ^2^ test, the comparative fit index (CFI ≥ 0.95), and the root mean square error of approximation (RMSEA ≤ 0.06) [70,71,72]. Measurement invariance was evaluated by comparing the configural (same factor structure), metric (equal factor loadings in addition to configural invariance), and scalar (equal item thresholds or intercepts in addition to metric invariance) invariance models. When a violation of invariance was found, the non-invariant items were located by examining the modification indices (MIs).

After examining the preliminary results of measurement invariance, we adopted the item parceling technique for subsequent analyses by averaging two to three items together as indicators for the latent variables to reduce the number of parameters and get more stable results [73,74,75]. Specifically, metric and scalar invariant items would only be combined with invariant items, and items that showed strong, unique factor covariance were more likely to be in the same parcel. For each psychological construct, partial invariance models, in which non-invariant indicators were allowed to be freely estimated across groups, were compared using both the original items and the item parcels to ensure that the latent factors had similar means and variances for each group. Information on how the item parcels were formed can be found in the Supplementary materials (Tables S2–S6).

#### 2.3.2. Latent Mean Comparisons, Latent Regressions, and Moderation Testing

Given that previous research has consistently shown the association between sex and IGD Tendency, sex differences on the path coefficients may be found in the predicting IGD Tendency; therefore, subsequent multiple-group SEM analyses were conducted with four groups: males and females from Japan, and males and females from China, so that differences in coefficients could be modeled. The latent factor means by sex and country were obtained using the previously formed item parcels with a CFA model (Model 1), including all latent factors for the psychological constructs with robust maximum likelihood estimation (MLR). Standardized mean differences (*d*) were reported, as well as the significance level(s) (*p* or *p*s). The means and standard deviations of the composite score of major variables by sex and country were provided in Table S7 in the Supplementary materials. To test H1, seven separate univariate latent regression models (Models 2–8), each with only (a) DASS-21 general and specific factors, (b) Mindfulness, (c) Coping Flexibility, and (d) Social Support, were performed to examine the bivariate relations. As an extended testing of H1, a latent multiple regression model (Model 9) was then fitted to examine whether these psychological variables were associated with IGD Tendency simultaneously. Finally, we examined the moderating effect of Mindfulness, Coping Flexibility, and Social Support on the association between DASS-21 and IGD Tendency (H2), using the latent moderated structural equations procedure in Mplus (Model 10) [76]. Standardized path coefficients (βs) were reported and interpreted in all the above-mentioned latent models.

## 3. Results

### 3.1. Measurement Invariance Testing and Item Parceling

As for the first study objective, results of the measurement invariance analyses showed some degree of non-invariance on some items for all five psychological constructs across countries and sexes; however, we were able to identify partial scalar invariance models with good model fit by freeing the constraints on the non-invariant items (with RMSEA = 0.036 to 0.061, CFI = 0.954 to 0.994). Detailed results for the measurement invariance analyses of each measure can be found in the Supplementary materials (Tables S1–S6).

With item parcels, the four-group CFA with correlated latent variables of IGD Tendency, DASS-21 (General, Depression, Anxiety, and Stress), Mindfulness, Coping Flexibility, and Social Support fitted the data well, χ^2^(1475) = 2126.62, RMSEA = 0.038, 90% CI [0.034, 0.041], CFI = 0.959, SRMR = 0.059. Because modification indices indicated scalar non-invariance for IGD Tendency (i.e., the intercepts of one parcel for the Japanese female group and another parcel for the Japanese male group), we further relaxed those invariance constraints and found an improved model fit, χ^2^(1473) = 2093.31, RMSEA = 0.037, 90% CI [0.033, 0.040], CFI = 0.961, SRMR = 0.059. Based on this modified measurement model, we computed the latent, age-adjusted means and standard deviations of the major variables (see Table 1) for subsequent cross-sample latent mean comparison.

### 3.2. Cross-Sample Latent Mean Comparison on IGD Tendency and Psychological Variables

Compared with their Chinese counterparts, Japanese participants reported significantly higher scores on IGD Tendency (*d* = 0.45, *p* < 0.001), DASS-21 General (*d* = 0.37, *p* = 0.009), and DASS-21 Depression (*d* = 0.47, *p* = 0.003), but significantly lower scores on DASS-21 Anxiety (*d* = 0.91, *p* < 0.001), Mindfulness (*d* = 0.23, *p* = 0.001), Coping Flexibility (*d* = 0.31, *p* < 0.001), and Social Support (*d* = 0.36, *p* < 0.001). Sex differences on the psychological variables had the exact directions and similar magnitudes for Japan and China, with males scoring significantly higher on IGD Tendency (*d* = 0.39 in Japan and 0.59 in China, *p*s ≤ 0.001) and Mindfulness (*d* = 0.20 in Japan and 0.19 in China, *p*s ≤ 0.044) and females scoring significantly higher on Social Support (*d* = 0.63 in Japan and 0.40 in China, *p*s < 0.001). In contrast, we did not find significant differences in age and years of study for IGD Tendency across samples (*p*s > 0.05).

### 3.3. Associations between IGD Tendency and Psychological Variables

For testing H1, we first entered each latent psychological variable independently in a series of latent regressions to explore their individual, bivariate association with IGD Tendency. As displayed in Table 2, across sexes and countries, IGD tendency was positively associated with DASS-21 General (β = 0.29, *p* < 0.001) and negatively associated with Mindfulness (β = −0.40, *p* < 0.001) and Social Support (β = −0.18, *p* < 0.001). No statistically significant associations were found for IGD Tendency with Coping Flexibility and DASS-21 specific factors (*p*s ≥ 0.24).

In the subsequent structural regression model, we simultaneously used DASS-21 factors, Mindfulness, Coping Flexibility, and Social Support to account for the variances in IGD Tendency as an extended testing of H1. As shown in Table 3, evidence for sex-specific coefficients of DASS-21 General on IGD Tendency were found, and the model fit was satisfactory, with χ^2^(1490) = 2107.20, RMSEA = 0.037, 90% CI [0.033, 0.040], CFI = 0.961, SRMR = 0.060. Specifically, in the same model, Mindfulness was found to be negatively associated with IGD Tendency across sexes, β = −0.33, *p* < 0.001, whereas DASS-21 General was positively associated with IGD for females, β = 0.20, *p* = 0.003, but not for males, β = 0.07, *p* = 0.38. The path coefficients for the specific factors of DASS-21, Coping Flexibility, and Social Support were not statistically significant (*p* > 0.14). Based on Model 9, the variance explained for IGD Tendency was 16.6% for males and 27.6% for females in Japan, and 18.3% for males and 23.8% for females in China.

### 3.4. Moderation Testing

We tested the two-way interactions between DASS-21 factors and three coping resources (i.e., DASS-21 General × Mindfulness, DASS-21 General × Coping Flexibility, and DASS-21 General × Social Support) based on the hypothesized moderation model (H2). When each interaction was tested separately, we found statistical evidence for DASS-21 General × Mindfulness, Δχ^2^(2) = 12.88, *p* = 0.002, but not for the other two interaction terms involving Coping Flexibility and Social Support. The pattern of the path coefficients suggested that the coefficient of DASS-21 General × Mindfulness differed across sexes but not country groups, with β = −0.06, *p* = 0.44, for males and β = −0.16, *p* = 0.002 for females (see Table 4).

**Figure 1:**
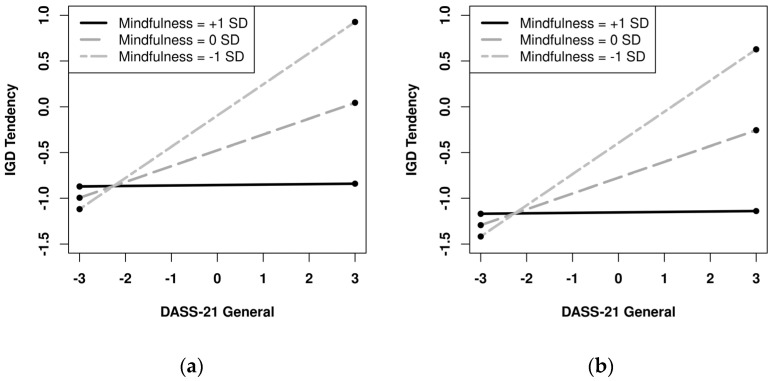
Interaction between DASS-21 General and Mindfulness on IGD Tendency for females in Japan (**a**) and China (**b**).

These results indicated that the moderating effect of DASS-21 General × Mindfulness on IGD tendency was only found in females from both countries, but not in males. We, hence, plotted the predicted linear relations between DASS-21 General and IGD Tendency for different levels of Mindfulness (i.e., +1 SD = high, 0 SD = medium, −1 SD = low) for females in both countries. As shown in Figure 1, in both Japanese and Chinese samples, for females with low Mindfulness, DASS-21 General and IGD Tendency showed a stronger relation; for females with high Mindfulness, DASS-21 General and IGD Tendency were only weakly related.

## 4. Discussion

Previous studies showed that Asian young people are more vulnerable to Internet-related addictions than their Western counterparts [66,77,78]. After confirming the measurement invariance of the measures across samples (Objective 1), this study found that Japanese university students reported a higher IGD tendency than Chinese students. This finding echoes Japanese university students’ higher Internet addiction vulnerability than Chinese university students in Yang et al.’s [12] study. Our Japanese student sample also reported significantly more psychological distress, particularly depression, than the Chinese sample. The univariate analysis results of this study showed that psychological distress had a significant positive correlation with IGD tendency in both Chinese and Japanese students. This finding was consistent with our hypothesis that psychological distress was a salient risk factor of IGD tendency. It also, at least partially, explained why our Japanese sample reported a higher IGD tendency.

Based on the transactional theory of stress and coping [30], this study was the first to investigate whether the three coping resources, namely, coping flexibility, mindfulness, and social support, are significant protective factors of IGD tendency among Japanese and Chinese university students. The univariate analyses showed that only mindfulness and social support, but not coping flexibility, were significantly associated with IGD tendency in a negative direction, lending partial support to H1. The expected protective effect of mindfulness and social support on IGD tendency corroborates the transactional theory of stress and coping, in which mindfulness and social support constitute imperative internal and external coping resources during the appraisal processes. As for the unexpected findings of coping flexibility, we inferred that its impact might be more direct with psychological distress and less direct with IGD, compared to the other two coping resources. Admittedly, in contrast to the relatively more established negative association between coping flexibility and psychological distress [79], the relationship between coping flexibility and IGD was not conclusive based on the dearth of extant empirical findings (i.e., [37]). Considering that we did not find the hypothesized moderating effect of coping flexibility in the link between psychological distress and IGD tendency either, more follow-up studies are encouraged to explore further the direct and indirect of coping flexibility and IGD, if any.

Our results identified both social support and mindfulness as protective factors against IGD vulnerability. The lower levels of mindfulness and social support reported by our Japanese students were attributed to be one of the reasons they were more vulnerable to IGD than their Chinese counterparts in this study. Concerning previous research (i.e., [80]), perceived social support can be promoted via various programs, such as a cost-effective peer-led intervention program among university students. Mindfulness not only emerged as the only significant protective factor against IGD in the multiple-group SEM analyses, but it also was found to significantly buffer the positive association between psychological distress and female students’ IGD tendency, partly in support of H2. Under the transactional theory of stress and coping, this buffering effect of mindfulness illustrates how gamers perceive their coping resources to surpass the internal demand of psychological distress and then take on more adaptive coping approaches other than excessive gaming. Such a buffering effect of mindfulness was also observed between Chinese working adults’ stress and their IGD tendency in a recent study [61]. In light of these findings, schools and local community centers involving Asian youth are recommended to consider mindfulness training programs to promote one’s mindful state/trait and stress/distress management [81,82] for preventing IGD. Given the shared philosophical roots of mindfulness and local cultures in Asian countries (e.g., Buddhism [83]), we expect the mindfulness training to be effectively incorporated into Chinese and Japanese societies, especially considering similar training has shown promising effects among Japanese and Chinese youth and adults [84,85,86], albeit the corresponding cost-effectiveness for IGD prevention needs to be evaluated further [87].

This study also identified some sex variations on associated factors of IGD tendency across Chinese and Japanese students. For example, the multiple-sample SEM (Model 9) found that, when considering other factors, psychological distress was positively associated with IGD for female students, but not for male students. Furthermore, the buffering effect of mindfulness was significant only among female students but not male students. Consistent with previous studies [88,89,90], our male students reported higher IGD tendencies across samples than our female counterparts. However, female students’ IGD susceptibility should not be neglected because they are psychologically vulnerable to IGD development, considering their higher levels of psychological distress and lower levels of mindful traits observed in this study. With the rapid increase of gaming among females and the associated change in social norms, female youth may become equally susceptible to IGD as their male counterparts are. Therefore, both sexes should be included in the school-based prevention programs, and the effects of those sex-specific risk/protective factors should be particularly noted if segmentation by sex is implemented.

Our study findings offered helpful insights for cross-cultural (in)variances on IGD associated factors, but they should be interpreted with caution because of some limitations in this study’s design. First, this is a cross-sectional study, and thus the causal relationship between the psychological variables and IGD tendency cannot be tested. Second, the data of the two student samples recruited by convenient sampling may not represent the general student populations and cannot be generalized to other populations (e.g., working adults) in China and Japan. Third, although the anonymous survey design should reduce the social desirability effect, the data were still susceptible to self-report bias; hence, we called for future studies to collect additional objective data, such as clinical inventories that evaluated participants’ behavioral responses, in order to provide a more comprehensive picture of the mind and its underlying mechanisms.

## 5. Conclusions

The high prevalence of psychological problems, such as depression, in Chinese and Japanese student populations has been a public concern [91,92,93,94], and its association with IGD has been consistently shown in this and previous research [25,95,96]. Through a series of statistical analyses, we first established the measurement invariance across countries and sexes for Chinese and Japanese gamers, paving the path for subsequent cross-cultural studies between these two countries. Our subsequent discovery of the between-country invariance in the associated risk and protective factors of IGD under the transactional theory of stress and coping points to promising directions of IGD prevention. Specifically, our findings suggest that some, but not all, factors of psychosocial resources for coping should be particularly considered and modified in school-based intervention programs for IGD in Asian students. Mindfulness was shown to be the most important coping resource, compared to coping flexibility and social support, for IGD; hence, we called for subsequent studies to further examine the cost-effectiveness of incorporating mindfulness training in school- or community-based IGD prevention programs. Sex differences must also be considered in designing such programs. Policymakers and health practitioners are advised to consider these between-country consistent findings when dealing with the potential hazards associated with IGD to maximize the policy and therapeutic influence. Scientific collaborations between China and Japan can also be strengthened to accelerate knowledge exchange and promotion as well as technology transformation to gaming industries (e.g., integrating mindfulness elements with games). Further longitudinal studies are needed to investigate the risk and protective effects of these identified psychosocial factors of IGD and empirically evaluate them in intervention programs with randomized controlled trials.

## Figures and Tables

**Table 1:** Means (age-adjusted) and standard deviations and comparison of the major variables in Model 1.

	Japan						China					
	Male (*n* = 265) ^a^		Female (*n* = 316)		Total (*n* = 581)		Male (*n* = 221)		Female (*n* = 441)		Total (*n* = 662)	
	*M*	*SD*	*M*	*SD*	*M*	*SD*	*M*	*SD*	*M*	*SD*	*M*	*SD*
IGD Tendency	0	1.00	−0.40	1.02	−0.22	1.01	−0.29	0.95	−0.83	0.90	−0.65	0.94
DASS-21 General	0	1.00	−0.10	1.01	−0.05	1.01	−0.30	1.07	−0.49	0.96	−0.43	1.04
DASS-21 Depression	0	1.00	−0.09	0.81	−0.05	0.92	−0.34	0.65	−0.43	0.38	−0.40	0.57
DASS-21 Anxiety	0	1.00	−0.29	0.64	−0.16	0.86	0.39	0.49	0.52	0.49	0.48	0.49
DASS-21 Stress	0	1.00	0.09	0.99	0.05	1.00	0.00	1.07	0.24	0.98	0.16	1.04
Mindfulness	0	1.00	−0.21	1.16	−0.12	1.08	0.27	1.11	0.07	1.02	0.14	1.08
Coping Flexibility	0	1.00	0.07	0.97	0.04	0.99	0.23	0.87	0.36	0.70	0.31	0.82
Social Support	0	1.00	0.62	0.98	0.34	0.99	0.45	0.75	0.76	0.77	0.65	0.76

^a^ The multiple-group structural equation model was identified by fixing the latent factor means to zero and the latent factor residual standard deviations to 1.0 for Japanese males. According to Kline [73], setting Japanese males as the reference in multi-group SEM only affects the scaling of the latent variables for the other three groups (i.e., Japanese females, Chinese males, and Chinese females), but not the differences across groups.

**Table 2:** Univariate latent regression models of IGD tendency across both sexes and two countries (Models 2 to 8).

Model	Path	β	*SE*	*p*
2	DASS-21 General → IGD Tendency	0.29	0.56	<0.001
3	DASS-21 Depression → IGD Tendency	0.08	0.11	0.45
4	DASS-21 Anxiety → IGD Tendency	0.08	0.10	0.40
5	DASS-21 Stress → IGD Tendency	0.01	0.06	0.88
6	Mindfulness → IGD Tendency	−0.40	0.05	<0.001
7	Coping Flexibility → IGD Tendency	−0.05	0.04	0.24
8	Social Support → IGD Tendency	−0.18	0.06	<0.001

Note: SE = standard error. The path coefficients were constrained equal across all four groups (i.e., Japanese males, Japanese females, Chinese males, and Chinese females).

**Table 3:** Latent multiple regression of IGD tendency across two countries (Model 9).

Antecedent		Male			Female	
	β	*SE*	*p*	β	*SE*	*p*
DASS-21 General	0.07	0.09	0.38	0.20	0.07	0.003
DASS-21 Depression	−0.02	0.11	0.85	0.23	0.18	0.17
DASS-21 Anxiety	0.03	0.12	0.80	−0.07	0.19	0.69
DASS-21 Stress	−0.13	0.10	0.14	0.01	0.08	0.87
Mindfulness	−0.33	0.06	<0.001	−0.33	0.06	<0.001
Coping Flexibility	0.00	0.05	0.94	0.00	0.05	0.94
Social Support	0.00	0.06	0.98	0.00	0.06	0.98

Note. SE = standard error. The model was adjusted for age. The path coefficients were constrained to be equal across countries as we found no evidence for between-country differences in them.

**Table 4:** Latent multiple regression of IGD tendency with moderating testing across two countries (Model 10).

Antecedent		Male			Female	
	β	*SE*	*p*	β	*SE*	*p*
DASS-21 General	0.04	0.09	0.60	0.18	0.09	0.005
DASS-21 Depression	0.07	0.09	0.36	0.07	0.09	0.36
DASS-21 Anxiety	−0.09	0.09	0.28	−0.09	0.09	0.28
DASS-21 Stress	−0.04	0.06	0.44	−0.04	0.06	0.44
Mindfulness	−0.39	0.07	<0.001	−0.39	0.07	<0.001
Coping Flexibility	0.00	0.05	0.93	0.00	0.05	0.93
Social Support	−0.01	0.06	0.86	−0.01	0.06	0.86
DASS-21 General x Mindfulness	−0.06	0.08	0.44	−0.16	0.06	0.002

Note. SE = standard error. The model was adjusted for age. The path coefficients were constrained to be equal across countries as we found no evidence for between-country differences in them.

## Data Availability

The data presented in this study are available on request from the corresponding author.

## Supplementary Materials

The following are available online at https://www.mdpi.com/article/10.3390/ijerph19052951/s1, Table S1: Model fit indices of the partial scalar invariance models across constructs/scales, Table S2: Measurement parameter estimates of the Internet Gaming Disorder Tendency, Table S3: Parameter estimates of a bifactor measurement model of the 21-item Depression Anxiety Stress Scales (DASS-21), Table S4: Measurement parameter estimates of the Mindful Attention Awareness Scale (MAAS), Table S5: Measurement parameter estimates of the Coping Flexibility Scale (CFS), Table S6: Parameter estimates of a bifactor measurement model of the Social Support Scale (SSS), Table S7: The means and standard deviations of the composite score of major variables by sex and country.
