# Supplementary material for: Effects of Psychological Distress and Coping Resources on Internet Gaming Disorder: Comparison between Chinese and Japanese University Students
Source: Int J Environ Res Public Health. 2022 Mar 3;19(5):2951. doi: 10.3390/ijerph19052951 (PMC8910164; doi:10.3390/ijerph19052951)
Supplement: Supplementary file 1 [file ijerph-19-02951-s001.zip › ijerph-1589395-supplementary.pdf]

Supplementary Materials (A): Model fit indices of the partial scalar invariance models across constructs/scales.

Table S1. Model fit indices of the partial scalar invariance models across constructs/scales.

	χ^2	*df*	*p*	CFI	RMSEA	RMSEA 90% CI
IGD Tendency	157.492	76	< 0.001	0.990	0.046	[0.035, 0.056]
DASS-21	993.893	403	< 0.001	0.980	0.049	[0.045, 0.052]
CFS	238.090	80	< 0.001	0.994	0.056	[0.048, 0.065]
MASS	674.389	204	< 0.001	0.954	0.061	[0.056, 0.066]
SSS	182.257	100	< 0.001	0.987	0.036	[0.028, 0.045]

Note. IGD = Internet Gaming Disorder. DASS-21 = 21-item Depression Anxiety Stress Scales. CFS = Coping Flexibility Scale. SSS = Social Support Scale. MAAS = Mindful Attention Awareness Scale. With the MLR estimator in Mplus, the χ^2 difference test was conducted using the Satorra–Bentler adjustment.

Supplementary Materials (B): Measurement parameters of the partial invariance model of the psychological constructs

Table S2. Measurement parameter estimates of the Internet Gaming Disorder Tendency.

Parcel	Item	Loading		Threshold 1		Threshold 2		Threshold 3		Threshold 4		Intercept	
		Japan	China	Japan	China	Japan	China	Japan	China	Japan	China	Japan	China
**1**	6	0.75 (0.03)		−0.22 (0.05)		0.52 (0.05)		1.16 (0.07)		1.87 (0.11)		0.00 (—)	
	8	0.60 (0.03)		−0.29 (0.05)		0.33 (0.05)		1.18 (0.07)		1.92 (0.11)		0.00 (—)	
**2**	7	0.65 (0.03)		0.32(0.05)		0.98 (0.06)		1.52 (0.08)		2.12 (0.12)		0.00 (—)	
	9	0.68 (0.03)		0.44 (0.05)		1.15 (0.07)		1.75 (0.10)		2.28 (0.16)		0.00 (—)	
**3**	1	0.72 (0.03)		−0.34 (0.06)	−0.72 (0.08)	0.41 (0.05)		1.38 (0.08)		2.09 (0.12)		0.00 (—)	
	2	0.76 (0.03)		0.00 (0.06)		0.91 (0.06)		1.84 (0.10)		2.55 (0.17)		0.00 (—)	0.30 (0.06)
	3	0.71 (0.03)		−0.01 (0.06)		0.83 (0.06)		1.59 (0.08)		2.17 (0.12)		0.00 (—)	0.36 (0.06)
**4**	4	0.78 (0.02)		−0.27 (0.06)		0.32 (0.06)	0.59 (0.07)	1.16 (0.07)		1.82 (0.11)		0.00 (—)	
	5	0.70 (0.03)	0.75 (0.08)	0.28 (0.06)		1.08 (0.07)		1.77 (0.11)		2.44 (0.17)		0.00 (—)	0.39 (0.06)

Note: Standard error estimates were shown in parentheses.

Table S3. Parameter estimates of a bifactor measurement model of the 21-item Depression Anxiety Stress Scales (DASS-21).

<table>
<tr><th rowspan="2">Parcel</th><th rowspan="2">Item</th><th colspan="2">General Loading</th><th colspan="2">Domain Loading</th><th colspan="2">Threshold 1</th><th colspan="2">Threshold 2</th><th colspan="2">Threshold 3</th><th colspan="2">Intercept</th></tr>
<tr><th>Japan</th><th>China</th><th>Japan</th><th>China</th><th>Japan</th><th>China</th><th>Japan</th><th>China</th><th>Japan</th><th>China</th><th>Japan</th><th>China</th></tr>
<tr><td rowspan="2">**D1**</td><td>Depression 1</td><td colspan="2">0.62 (0.03)</td><td colspan="2">0.58 (0.03)</td><td colspan="2">−0.12 (0.05)</td><td colspan="2">0.84 (0.05)</td><td colspan="2">1.67 (0.08)</td><td colspan="2">0.00 (—)</td></tr>
<tr><td>Depression 4</td><td colspan="2">0.61 (0.03)</td><td colspan="2">0.53 (0.03)</td><td colspan="2">−0.25 (0.05)</td><td colspan="2">0.68 (0.05)</td><td colspan="2">1.38 (0.07)</td><td colspan="2">0.00 (—)</td></tr>
<tr><td rowspan="3">**D2**</td><td>Depression 5</td><td colspan="2">0.49 (0.03)</td><td colspan="2">0.60 (0.03)</td><td colspan="2">−0.25 (0.05)</td><td colspan="2">0.66 (0.05)</td><td colspan="2">1.47 (0.07)</td><td colspan="2">0.00 (—)</td></tr>
<tr><td>Depression 6</td><td colspan="2">0.50 (0.03)</td><td colspan="2">0.76 (0.02)</td><td colspan="2">0.18 (0.05)</td><td colspan="2">0.87 (0.05)</td><td colspan="2">1.49 (0.07)</td><td colspan="2">0.00 (—)</td></tr>
<tr><td>Depression 7</td><td colspan="2">0.51 (0.03)</td><td colspan="2">0.76 (0.03)</td><td colspan="2">0.28 (0.05)</td><td colspan="2">0.97 (0.06)</td><td colspan="2">1.55 (0.08)</td><td colspan="2">0.00 (—)</td></tr>
<tr><td rowspan="2">**D3**</td><td>Depression 2</td><td colspan="2">0.48 (0.03)</td><td colspan="2">0.52 (0.03)</td><td colspan="2">−0.47 (0.05)</td><td colspan="2">0.59 (0.05)</td><td colspan="2">1.47 (0.08)</td><td colspan="2">0.00 (—)</td></tr>
<tr><td>Depression 3</td><td colspan="2">0.53 (0.03)</td><td colspan="2">0.65 (0.03)</td><td colspan="2">−0.13 (0.05)</td><td colspan="2">0.76 (0.05)</td><td>1.66 (0.09)</td><td>1.18 (0.09)</td><td colspan="2">0.00 (—)</td></tr>
<tr><td rowspan="2">**A1**</td><td>Anxiety 1</td><td colspan="2">0.41 (0.03)</td><td colspan="2">0.30 (0.04)</td><td colspan="2">−0.48 (0.05)</td><td colspan="2">0.66 (0.05)</td><td colspan="2">1.54 (0.08)</td><td>0.00 (—)</td><td>−0.22 (0.07)</td></tr>
<tr><td>Anxiety 7</td><td colspan="2">0.59 (0.03)</td><td colspan="2">0.68 (0.03)</td><td colspan="2">0.45 (0.05)</td><td colspan="2">1.17 (0.06)</td><td colspan="2">1.66 (0.07)</td><td colspan="2">0.00 (—)</td></tr>
<tr><td rowspan="3">**A2**</td><td>Anxiety 3</td><td colspan="2">0.38 (0.03)</td><td colspan="2">0.69 (0.03)</td><td colspan="2">0.56 (0.05)</td><td colspan="2">1.26 (0.06)</td><td colspan="2">1.81 (0.09)</td><td colspan="2">0.00 (—)</td></tr>
<tr><td>Anxiety 4</td><td colspan="2">0.55 (0.03)</td><td colspan="2">0.49 (0.03)</td><td colspan="2">−0.23 (0.05)</td><td colspan="2">0.58 (0.05)</td><td colspan="2">1.33 (0.06)</td><td colspan="2">0.00 (—)</td></tr>
<tr><td>Anxiety 5</td><td colspan="2">0.57 (0.03)</td><td colspan="2">0.56 (0.03)</td><td colspan="2">0.44 (0.05)</td><td colspan="2">1.11 (0.05)</td><td colspan="2">1.70 (0.08)</td><td colspan="2">0.00 (—)</td></tr>
<tr><td rowspan="2">**A3**</td><td>Anxiety 2</td><td colspan="2">0.47 (0.03)</td><td colspan="2">0.70 (0.03)</td><td colspan="2">0.49 (0.06)</td><td colspan="2">1.18 (0.06)</td><td colspan="2">1.98 (0.10)</td><td colspan="2">0.00 (—)</td></tr>
<tr><td>Anxiety 6</td><td colspan="2">0.51 (0.03)</td><td colspan="2">0.70 (0.03)</td><td colspan="2">0.27 (0.05)</td><td colspan="2">1.01 (0.06)</td><td colspan="2">1.70 (0.08)</td><td>0.00 (—)</td><td>−0.30 (0.06)</td></tr>
<tr><td rowspan="2">**S1**</td><td>Stress 1</td><td colspan="2">0.71 (0.03)</td><td colspan="2">0.16 (0.05)</td><td colspan="2">−0.75 (0.05)</td><td colspan="2">0.57 (0.05)</td><td colspan="2">1.42 (0.07)</td><td colspan="2">0.00 (—)</td></tr>
<tr><td>Stress 5</td><td colspan="2">0.83 (0.03)</td><td colspan="2">0.16 (0.05)</td><td colspan="2">−0.71 (0.05)</td><td colspan="2">0.36 (0.05)</td><td colspan="2">1.29 (0.06)</td><td colspan="2">0.00 (—)</td></tr>
<tr><td rowspan="2">**S2**</td><td>Stress 6</td><td colspan="2">0.66 (0.03)</td><td colspan="2">−0.10 (0.05)</td><td colspan="2">−0.85 (0.06)</td><td colspan="2">0.44 (0.05)</td><td colspan="2">1.48 (0.07)</td><td colspan="2">0.00 (—)</td></tr>
<tr><td>Stress 7</td><td colspan="2">0.69 (0.03)</td><td colspan="2">−0.28 (0.06)</td><td colspan="2">−0.53 (0.06)</td><td colspan="2">0.56 (0.05)</td><td colspan="2">1.47 (0.07)</td><td colspan="2">0.00 (—)</td></tr>
<tr><td rowspan="3">**S3**</td><td>Stress 2</td><td colspan="2">0.68 (0.03)</td><td colspan="2">0.38 (0.05)</td><td colspan="2">−1.08 (0.06)</td><td>0.07 (0.05)</td><td>0.45 (0.07)</td><td colspan="2">1.32 (0.07)</td><td colspan="2">0.00 (—)</td></tr>
<tr><td>Stress 3</td><td colspan="2">0.68 (0.04)</td><td colspan="2">0.60 (0.05)</td><td colspan="2">−1.07 (0.06)</td><td colspan="2">−0.01 (0.05)</td><td>1.22 (0.07)</td><td>0.74 (0.09)</td><td colspan="2">0.00 (—)</td></tr>
<tr><td>Stress 4</td><td colspan="2">0.72 (0.03)</td><td colspan="2">0.35 (0.05)</td><td>−1.31 (0.07)</td><td>−0.73 (0.06)</td><td>−0.21 (0.05)</td><td>0.21 (0.05)</td><td colspan="2">0.88 (0.06)</td><td colspan="2">0.00 (—)</td></tr>
</table>

Note: Standard error estimates were shown in parentheses. A bifactor models was fitted with a general (Psychological Distress for DASS-21) and three specific factors (Depression, Anxiety, and Stress for DASS-21), based on overall improvement in fit and previous literature[1].

[1] For example, Gomez, R.; Summers, M.; Summers, A.; Wolf, A.; Summers, J. Depression Anxiety Stress Scales-21: Measurement and Structural Invariance Across Ratings of Men and Women. Assessment 2014, 21, 418–426. https://doi.org/10.1177/1073191113514106.

Table S4. Measurement parameter estimates of the Mindful Attention Awareness Scale (MAAS).

<table>
<tr><th rowspan="2">Parcel</th><th rowspan="2">Item</th><th colspan="2">Loading</th><th colspan="2">Threshold 1</th><th colspan="2">Threshold 2</th><th colspan="2">Threshold 3</th><th colspan="2">Threshold 4</th><th colspan="2">Threshold 5</th><th colspan="2">Intercept</th></tr>
<tr><th>Japan</th><th>China</th><th>Japan</th><th>China</th><th>Japan</th><th>China</th><th>Japan</th><th>China</th><th>Japan</th><th>China</th><th>Japan</th><th>China</th><th>Japan</th><th>China</th></tr>
<tr><td rowspan="2">1</td><td>6</td><td colspan="2">0.52 (0.03)</td><td colspan="2">−1.14 (0.06)</td><td colspan="2">−0.18 (0.04)</td><td colspan="2">0.46 (0.04)</td><td colspan="2">0.96 (0.05)</td><td colspan="2">1.44 (0.07)</td><td colspan="2">0.00 (—)</td></tr>
<tr><td>14</td><td colspan="2">0.56 (0.02)</td><td colspan="2">−1.40 (0.06)</td><td colspan="2">−0.36 (0.04)</td><td colspan="2">0.38 (0.04)</td><td colspan="2">0.96 (0.05)</td><td colspan="2">1.59 (0.07)</td><td colspan="2">0.00 (—)</td></tr>
<tr><td rowspan="2">2</td><td>9</td><td colspan="2">0.60 (0.02)</td><td colspan="2">−1.08 (0.05)</td><td colspan="2">−0.08 (0.04)</td><td colspan="2">0.72 (0.05)</td><td colspan="2">1.35 (0.06)</td><td colspan="2">1.91 (0.09)</td><td colspan="2">0.00 (—)</td></tr>
<tr><td>10</td><td colspan="2">0.70 (0.02)</td><td colspan="2">−0.90 (0.05)</td><td colspan="2">−0.01 (0.04)</td><td colspan="2">0.71 (0.05)</td><td colspan="2">1.44 (0.06)</td><td colspan="2">1.93 (0.09)</td><td colspan="2">0.00 (—)</td></tr>
<tr><td rowspan="4">3</td><td>1</td><td>0.48 (0.03)</td><td>0.38 (0.04)</td><td>−1.00 (0.06)</td><td>−1.38 (0.07)</td><td>0.11 (0.05)</td><td>−0.28 (0.05)</td><td>0.75 (0.06)</td><td>0.65 (0.06)</td><td>1.50 (0.08)</td><td>1.55 (0.08)</td><td>2.38 (0.16)</td><td>2.02 (0.11)</td><td colspan="2">0.00 (—)</td></tr>
<tr><td>2</td><td>0.56 (0.03)</td><td>0.48 (0.04)</td><td>−1.28 (0.07)</td><td>−1.04 (0.06)</td><td>−0.26 (0.05)</td><td>−0.00 (0.05)</td><td>0.43 (0.05)</td><td>0.74 (0.06)</td><td>1.12 (0.07)</td><td>1.29 (0.07)</td><td>1.63 (0.09)</td><td>1.90 (0.10)</td><td colspan="2">0.00 (—)</td></tr>
<tr><td>4</td><td>0.40 (0.04)</td><td>0.55 (0.04)</td><td>−0.73 (0.06)</td><td>−1.25 (0.07)</td><td>0.22 (0.05)</td><td>−0.21 (0.05)</td><td>0.67 (0.06)</td><td>0.57 (0.06)</td><td>1.17 (0.07)</td><td>1.21 (0.07)</td><td>1.80 (0.10)</td><td>1.70 (0.09)</td><td colspan="2">0.00 (—)</td></tr>
<tr><td>5</td><td colspan="2">0.43 (0.03)</td><td colspan="2">−0.69 (0.06)</td><td colspan="2">0.20 (0.05)</td><td colspan="2">0.85 (0.05)</td><td colspan="2">1.37 (0.07)</td><td colspan="2">1.91 (0.10)</td><td>0.00 (—)</td><td>0.21 (0.06)</td></tr>
<tr><td rowspan="3">4</td><td>7</td><td colspan="2">0.74 (0.02)</td><td colspan="2">−1.39 (0.07)</td><td colspan="2">−0.37 (0.05)</td><td colspan="2">0.36 (0.05)</td><td colspan="2">1.07 (0.06)</td><td colspan="2">1.69 (0.08)</td><td>0.00 (—)</td><td>−0.25 (0.05)</td></tr>
<tr><td>8</td><td>0.60 (0.03)</td><td>0.74 (0.04)</td><td>−1.03 (0.06)</td><td>−1.38 (0.07)</td><td>−0.05 (0.05)</td><td>−0.20 (0.06)</td><td>0.59 (0.06)</td><td>0.62 (0.06)</td><td>1.23 (0.07)</td><td>1.31 (0.08)</td><td>1.84 (0.10)</td><td>1.92 (0.11)</td><td colspan="2">0.00 (—)</td></tr>
<tr><td>11</td><td>0.49 (0.03)</td><td>0.42 (0.04)</td><td>−1.70 (0.09)</td><td>−1.58 (0.08)</td><td>−0.28 (0.05)</td><td>−0.45 (0.05)</td><td>0.36 (0.05)</td><td>0.41 (0.06)</td><td>1.18 (0.07)</td><td>1.13 (0.07)</td><td>1.95 (0.11)</td><td>1.77 (0.10)</td><td colspan="2">0.00 (—)</td></tr>
<tr><td rowspan="4">5</td><td>3</td><td colspan="2">0.56 (0.02)</td><td colspan="2">−1.47 (0.07)</td><td colspan="2">−0.36 (0.05)</td><td colspan="2">0.41 (0.05)</td><td colspan="2">1.08 (0.06)</td><td colspan="2">1.69 (0.08)</td><td>0.00 (—)</td><td>−0.29 (0.06)</td></tr>
<tr><td>12</td><td colspan="2">0.55 (0.03)</td><td>0.33 (0.05)</td><td>−0.06 (0.06)</td><td colspan="2">0.73 (0.05)</td><td colspan="2">1.30 (0.06)</td><td colspan="2">1.91 (0.09)</td><td colspan="2">2.46 (0.14)</td><td colspan="2">0.00 (—)</td></tr>
<tr><td>13</td><td>0.28 (0.04)</td><td>0.51 (0.04)</td><td>−1.95 (0.11)</td><td>−1.59 (0.08)</td><td>−1.02 (0.06)</td><td>−0.55 (0.06)</td><td>−0.46 (0.05)</td><td>0.04 (0.05)</td><td>0.25 (0.05)</td><td>0.76 (0.06)</td><td>0.96 (0.06)</td><td>1.36 (0.08)</td><td colspan="2">0.00 (—)</td></tr>
<tr><td>15</td><td>0.53 (0.04)</td><td>0.38 (0.04)</td><td>−0.23 (0.05)</td><td>−0.66 (0.05)</td><td>0.36 (0.05)</td><td>0.12 (0.05)</td><td>0.73 (0.06)</td><td>0.64 (0.06)</td><td>1.23 (0.07)</td><td>1.17 (0.07)</td><td>1.72 (0.09)</td><td>1.62 (0.08)</td><td colspan="2">0.00 (—)</td></tr>
</table>

Note: Standard error estimates were shown in parentheses.

Table S5. Measurement parameter estimates of the Coping Flexibility Scale (CFS).

<table>
<tr><th rowspan="2">Parcel</th><th rowspan="2">Item</th><th colspan="2">Loading</th><th colspan="2">Threshold 1</th><th colspan="2">Threshold 2</th><th colspan="2">Threshold 3</th><th colspan="2">Intercept</th></tr>
<tr><th>Japan</th><th>China</th><th>Japan</th><th>China</th><th>Japan</th><th>China</th><th>Japan</th><th>China</th><th>Japan</th><th>China</th></tr>
<tr><td rowspan="2">1</td><td>4</td><td colspan="2">0.89 (0.01)</td><td colspan="2">−1.50 (0.07)</td><td colspan="2">−0.48 (0.05)</td><td colspan="2">1.14 (0.06)</td><td colspan="2">0.00 (—)</td></tr>
<tr><td>5</td><td colspan="2">0.95 (0.01)</td><td colspan="2">−1.65 (0.08)</td><td colspan="2">−0.54 (0.05)</td><td colspan="2">1.20 (0.07)</td><td colspan="2">0.00 (—)</td></tr>
<tr><td rowspan="2">2</td><td>8</td><td colspan="2">0.93 (0.01)</td><td colspan="2">−1.65 (0.08)</td><td colspan="2">−0.46 (0.05)</td><td colspan="2">1.28 (0.07)</td><td colspan="2">0.00 (—)</td></tr>
<tr><td>10</td><td colspan="2">0.91 (0.01)</td><td colspan="2">−1.60 (0.08)</td><td colspan="2">−0.47 (0.05)</td><td colspan="2">1.16 (0.07)</td><td colspan="2">0.00 (—)</td></tr>
<tr><td rowspan="3">3</td><td>2</td><td colspan="2">0.01 (0.03)</td><td colspan="2">−1.14 (0.07)</td><td colspan="2">0.33 (0.05)</td><td colspan="2">1.72 (0.08)</td><td>0.00 (—)</td><td>0.32 (0.07)</td></tr>
<tr><td>6</td><td>0.53 (0.03)</td><td>0.77 (0.05)</td><td>−1.47 (0.08)</td><td>−1.59 (0.10)</td><td>−0.26 (0.05)</td><td>−0.40 (0.06)</td><td>1.18 (0.07)</td><td>1.39 (0.07)</td><td colspan="2">0.00 (—)</td></tr>
<tr><td>7</td><td colspan="2">−0.14 (0.03)</td><td colspan="2">−1.25 (0.07)</td><td colspan="2">0.06 (0.05)</td><td colspan="2">1.27 (0.07)</td><td>0.00 (—)</td><td>−0.39 (0.06)</td></tr>
<tr><td rowspan="3">4</td><td>1</td><td>0.69 (0.02)</td><td>0.83 (0.04)</td><td>−1.68 (0.09)</td><td>−1.58 (0.10)</td><td>−0.38 (0.05)</td><td>−0.71(0.07)</td><td>1.26 (0.07)</td><td>0.90 (0.06)</td><td colspan="2">0.00 (—)</td></tr>
<tr><td>3</td><td>0.75 (0.02)</td><td>0.89 (0.05)</td><td>−1.57 (0.08)</td><td>−1.72 (0.11)</td><td>−0.41 (0.05)</td><td>−0.39 (0.06)</td><td>1.19 (0.07)</td><td>1.01 (0.06)</td><td colspan="2">0.00 (—)</td></tr>
<tr><td>9</td><td>0.42 (0.04)</td><td>0.63 (0.05)</td><td colspan="2">−0.94 (0.06)</td><td colspan="2">0.18 (0.05)</td><td colspan="2">1.55 (0.08)</td><td>0.00 (—)</td><td>0.51 (0.06)</td></tr>
</table>

Note: Standard error estimates were shown in parentheses.

Table S6. Parameter estimates of a bifactor measurement model of the Social Support Scale (SSS).

<table>
<tr><th rowspan="2">Parcel</th><th rowspan="2">Item</th><th rowspan="2">Domain</th><th colspan="2">General Loading</th><th colspan="2">Domain Loading</th><th colspan="2">Intercept</th></tr>
<tr><th>Japan</th><th>China</th><th>Japan</th><th>China</th><th>Japan</th><th>China</th></tr>
<tr><td rowspan="2">1</td><td>1</td><td rowspan="4">Significant Others</td><td colspan="2">0.83 (0.04)</td><td colspan="2">0.58 (0.10)</td><td colspan="2">3.35 (0.05)</td></tr>
<tr><td>2</td><td colspan="2">0.91 (0.04)</td><td colspan="2">0.42 (0.09)</td><td colspan="2">3.55 (0.05)</td></tr>
<tr><td rowspan="2">2</td><td>5</td><td>0.94 (0.04)</td><td>0.74 (0.05)</td><td colspan="2">0.23 (0.08)</td><td colspan="2">3.70 (0.05)</td></tr>
<tr><td>10</td><td colspan="2">0.96 (0.04)</td><td colspan="2">0.04 (0.07)</td><td colspan="2">3.74 (0.05)</td></tr>
<tr><td rowspan="2">3</td><td>4</td><td rowspan="4">Family</td><td colspan="2">0.63 (0.04)</td><td>0.62 (0.06)</td><td>0.89 (0.08)</td><td>3.72 (0.05)</td><td>3.89 (0.06)</td></tr>
<tr><td>8</td><td colspan="2">0.71 (0.04)</td><td colspan="2">0.80 (0.06)</td><td>3.72 (0.05)</td><td>3.88 (0.06)</td></tr>
<tr><td rowspan="2">4</td><td>3</td><td colspan="2">0.63 (0.04)</td><td colspan="2">0.65 (0.05)</td><td colspan="2">3.96 (0.04)</td></tr>
<tr><td>11</td><td colspan="2">0.46 (0.04)</td><td colspan="2">0.48 (0.04)</td><td>2.69 (0.05)</td><td>3.78 (0.05)</td></tr>
<tr><td rowspan="2">5</td><td>6</td><td rowspan="4">Friends</td><td colspan="2">0.82 (0.03)</td><td colspan="2">0.40 (0.05)</td><td colspan="2">3.76 (0.04)</td></tr>
<tr><td>7</td><td colspan="2">0.83 (0.04)</td><td colspan="2">0.49 (0.05)</td><td colspan="2">3.68 (0.04)</td></tr>
<tr><td rowspan="2">6</td><td>9</td><td colspan="2">0.84 (0.04)</td><td colspan="2">0.45 (0.06)</td><td colspan="2">3.82 (0.04)</td></tr>
<tr><td>12</td><td colspan="2">0.82 (0.04)</td><td colspan="2">0.52 (0.05)</td><td colspan="2">3.83 (0.04)</td></tr>
</table>

Note: Standard error estimates were shown in parentheses. For SSS, WLSMV did not lead to converged results. Instead, we evaluated the measurement model for SSS using robust maximum likelihood estimation (ESTIMATOR = MLR in Mplus). A bifactor model was fitted with a general (i.e., General Social Support) and three specific factors (i.e., Family, Friends, and Significant Others), based on overall improvement in fit and previous literature[2]. However, given that most of the variance in SSS could respectively be explained by a common dimension (ω_H = .83 to .84 across samples), we only used the general SSS factor but not the specific factors for family, friends, and significant others in subsequent analyses.

[2] For example, Osman, A.; Lamis, D.A.; Freedenthal, S.; Gutierrez, P.M.; McNaughton−Cassill, M. The Multidimensional Scale of Perceived Social Support: Analyses of Internal Reliability, Measurement Invariance, and Correlates Across Gender. *Journal of Personality Assessment* **2014**, *96*, 103–112. https://doi.org/10.1080/00223891.2013.838170.

Supplementary Materials (C): The means and standard deviations of the composite score of major variables by sex and country

Table S7. The means and standard deviations of the composite score of major variables by sex and country.

	Japan						China					
	Male (n = 265)		Female (n = 316)		Total (*n* = 581)		Male (*n* = 221)		Female (*n* = 441)		Total (*n* = 662)	
	M	*SD*	*M*	*SD*	*M*	*SD*	*M*	*SD*	*M*	*SD*	*M*	*SD*
IGD Tendency	1.93	0.67	1.76	0.67	1.84	0.67	2.01	0.63	1.68	0.58	1.79	0.62
DASS-21 General	0.97	0.57	0.89	0.54	0.93	0.56	0.78	0.53	0.72	0.49	0.74	0.50
DASS-21 Depression	0.93	0.62	0.83	0.56	0.88	0.59	0.71	0.56	0.65	0.52	0.67	0.53
DASS-21 Anxiety	1.04	0.59	0.99	0.58	1.01	0.59	0.73	0.55	0.67	0.49	0.69	0.51
DASS-21 Stress	0.94	0.60	0.84	0.58	0.89	0.59	0.90	0.57	0.85	0.54	0.87	0.55
Mindfulness	4.25	0.66	4.10	0.75	4.17	0.71	4.35	0.71	4.21	0.67	4.26	0.68
Coping Flexibility	2.67	0.48	2.66	0.46	2.66	0.47	2.83	0.46	2.89	0.41	2.87	0.43
Social Support	3.39	0.80	3.83	0.79	3.63	0.83	3.75	0.67	4.00	0.63	3.92	0.65

Note. IGD = Internet Gaming Disorder. DASS-21 = 21-item Depression Anxiety Stress Scales. The numbers presented in this table are based on the composite scores (i.e., the arithmetic mean of its corresponding variable), which did not adjust for violations of measurement invariance. For some variables, the group means of the composite scores shown in this table follow a different ordering from that of the latent means in Table 1 (see the manuscript), mainly due to (a) age adjustment, (b) the latent means using optimized weights of items based on the factor loadings, and (c) the latent means also adjusting for measurement non-invariance. Please refer to Table 1 in the manuscript for more accurate comparisons across groups based on the age-adjusted latent means.
